# Cerebrospinal fluid neurofilament light chain in multiple sclerosis and its subtypes: a meta-analysis of case–control studies

**DOI:** 10.1136/jnnp-2018-319190

**Published:** 2019-05-23

**Authors:** Sarah-Jane Martin, Sarah McGlasson, David Hunt, James Overell

**Affiliations:** 1 Anne Rowling Centre for Regenerative Neurology, University of Edinburgh, Edinburgh, UK; 2 University of Glasgow, Glasgow, UK; 3 MRC Institute of Genetics and Molecular Medicine, University of Edinburgh, Edinburgh, UK; 4 Centre for Clinical Brain Sciences, University of Edinburgh, Edinburgh, UK; 5 Glasgow Multiple Sclerosis Clinical Research Centre, Queen Elizabeth University Hospital, Glasgow, UK

**Keywords:** multiple sclerosis, CSF, neuroimmunology

## Abstract

**Objective:**

Neurofilament is a biomarker of axonal injury proposed as a useful adjunct in the monitoring of patients with multiple sclerosis (MS). We conducted a systematic review and meta-analysis of case–control studies that have measured neurofilament light chain (NfL) levels in cerebrospinal fluid (CSF) of people with MS (pwMS), in order to determine whether, and to what degree, CSF NfL levels differentiate MS from controls, or the subtypes or stages of MS from each other.

**Methods:**

Guidelines on Preferred Reporting Items for Systematic Reviews and Meta-Analyses were followed. Electronic databases were searched for published and ‘grey’ literature, with 151 hits. Of 51 full articles screened, 20 were included in qualitative analysis, and 14 in meta-analysis.

**Results:**

CSF NfL was higher in 746 pwMS than 435 (healthy and disease) controls, with a moderate effect size of 0.61 (p < 0.00001). Mean CSF NfL levels were significantly higher in 176 pwMS with relapsing disease than 92 with progressive disease (2124.8 ng/L, SD 3348.9 vs 1121.4 ng/L, SD 947.7, p = 0.0108). CSF NfL in 138 pwMS in relapse (irrespective of MS subtype) was double that seen in 268 pwMS in remission (3080.6 ng/L, SD 4715.9 vs 1541.7 ng/L, SD 2406.5, p < 0.0001).

**Conclusions:**

CSF NfL correlates with MS activity throughout the course of MS, reflecting the axonal damage in pwMS. Relapse is more strongly associated with elevated CSF NfL levels than the development of progression, and NfL may be most useful as a marker of disease ‘activity’ rather than as a marker of disability or disease stage.

## Introduction

Therapy for multiple sclerosis (MS) has expanded remarkably over the past 20 years. The updated 2017 McDonald diagnostic criteria enable diagnosis and disease-modifying treatment (DMT) to occur earlier, and also recommend the diagnostic use of cerebrospinal fluid oligoclonal bands (CSF OCBs), which may now act as a proxy for evidence of dissemination in time.^1^ Prior to this revision the routine clinical use of fluid biomarkers had changed little since OCBs were implemented in the 1980s.

MRI remains the most commonly employed diagnostic and monitoring tool.^2^ Lesion location and burden are used as a prognostic aid, and once diagnosed, patients undergo regular imaging to assess white matter lesion load and monitor for adverse effects of DMT.^3^ However, standard MRI sequences do not fully reflect the scope of disease pathology, and correlation between MRI measures and clinical disability remains limited.^4–7^ It is now accepted that MS is not only an inflammatory disease of white matter but also grey matter, and that neurodegeneration occurs early in the disease process, and not merely as a consequence of demyelination.^8^ Pathological studies demonstrate diffuse axonal damage throughout normal appearing white matter—findings that have been replicated in vivo using MR spectroscopy, but the extent of diffuse white matter pathology does not correlate with the number of focal lesions on routine MRI sequences.^8 9^ General and regional MRI atrophy measures can reflect neurodegeneration to a degree, and correlate with longer-term measures of clinical disability, but are problematic to employ in standard practice in individual patients.^10 11^ Consequently, there is a need for a practical biomarker to quantify and monitor neurodegeneration.

Neurofilament is an intermediate filament protein, integral for radial growth of axons during development, and the cytoarchitecture and transport functions within mature neuronal axons.^12^ Axonal injury releases neurofilament into the extracellular space, where neurofilament light chain (NfL) can be measured in CSF as a biomarker of axonal degeneration. Optimised assays, and now the ability to measure NfL levels in blood, have increased its potential for translation to clinical practice.^13–18^ However, MS is a heterogeneous disease, with subtypes and trajectories, in flux between relapse, remission, stability and progression.^19^ Age, gender and comorbidities can influence any potential biomarker, and DMTs (with differing mechanisms of action) are widely prescribed. Disparities within measures used to classify the disease (disability scales, clinical staging and MRI) add a further hurdle in standardisation. In order to account for predictable variability, some aspects can be standardised.^20 21^ Where this is not possible, large datasets and repeated validations are required.

To improve the power to assess the relevance and utility of NfL measurement, we performed a systematic review and meta-analysis of CSF NfL in MS.

## Methods

### Objective

The focused question was ‘To what extent do CSF NfL levels differentiate people with MS (pwMS) from (healthy or disease) controls?’ Thereafter, ‘Can CSF NfL levels differentiate different MS disease stages or states’? The meta-analysis was registered with PROSPERO (ID CRD42017078996) and conducted according to a predetermined protocol.

### Selection criteria

Any original study quantifying NfL in CSF of pwMS was identified. No language or publication date restrictions were imposed. Patients of any age were included, with no restrictions on disease duration or subtype, time since relapse, disability, comorbidities or treatment.

Diagnosis had to be stated with reference to established diagnostic criteria. Where cohorts were not differentiated by MS subtype, they were named accordingly, for example ‘clinically definite MS (CDMS)’.^22 23^ Clinical and radiologically isolated syndromes were excluded in order to reduce the heterogeneity of the overall cohort. Each MS cohort required a control comparator. Ideally, studies should reference guidelines on defining control groups, but this was not an inclusion criterion.^21^ Studies could be retrospective, cross-sectional or prospective.

CSF collection and bio-banking were required to meet criteria proposed by BioMS-EU.^20^ If these criteria were not referenced, the paper was required to describe CSF sampling, pre-analytical handling and storage techniques applied to ensure the samples used were of sufficient quality. Studies also had to use a validated assay, or describe the ELISA technique to satisfy inclusion. Assays with a coefficient of variation >25% were excluded, as were studies where NfL was detectable in less than 85% of either comparator group.

### Search strategy for identification of studies and methods of review

**Figure 1:**
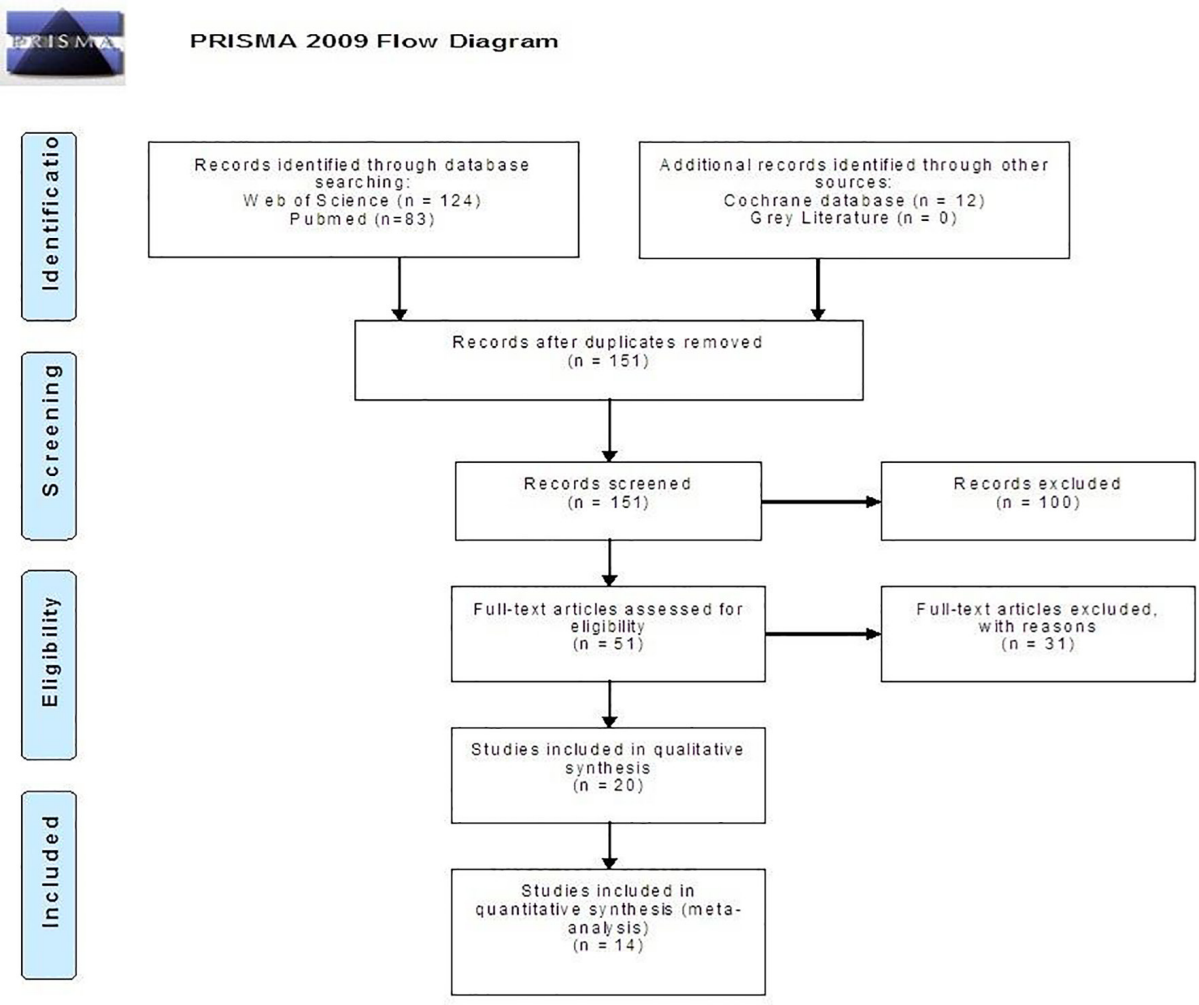
PRISMA 2009 flow diagram. PRISMA, Preferred Reporting Items for Systematic Review and Meta-analysis.

One author (SJM) searched electronic databases for published and unpublished ‘grey’ literature (online supplementary table 1), and reviewed abstracts to assess if they met inclusion criteria (online supplementary table 2). Detailed review of potentially eligible papers followed, as per Preferred Reporting Items for Systematic Reviews and Meta-Analyses, 2009 guidelines (figure 1).^24^

10.1136/jnnp-2018-319190.supp8 Supplementary data

### Description of studies and reasons for exclusion

In all, 68 duplicates were removed, and 100 records excluded on abstract alone (online supplementary table 3). Of the remaining 51 papers, 17 studies had no control group, three measured NfL in a categorical way and one measured serum NfL levels. Seven studies were excluded on the basis that CSF NfL level was detectable in less than 85% of one comparator group. These were older studies that used a less sensitive assay.^25 26^ Three papers were excluded as NfL levels for the MS cohort had previously been published. One paper used previously published data from a control cohort, but compared it with a new MS cohort, and was included.^27^ In all, 20 studies met inclusion criteria.

### Data extraction

Data were reported as mean and SD in some papers, and as median and range or IQR in others. If data were not provided as mean and SD, the authors were contacted and asked to provide these values or the raw data. Authors of six studies without the required information did not respond to requests for data, leaving 14 studies for analysis.

### Description of studies

In total, 805 patients (638 relapsing remitting MS (RRMS), 104 secondary progressive MS (SPMS) and 63 primary progressive MS (PPMS)) and 435 controls (332 non-inflammatory neurological disease controls (NINDCs) and 103 healthy controls (HCs)) from 14 studies were included (online supplementary figure 1). The Newcastle-Ottawa Scale (NOS—a scoring system to assist with quality assessment of non-randomised research) was implemented (table 1). However, as the NOS has not been validated no articles were excluded based on this score.^28^

10.1136/jnnp-2018-319190.supp1 Supplementary data

**Table 1:** Summary table of studies included in meta-analysis

First author, year	No MS	Mean age patients	No controls	Mean age controls	Quality (NOS)		
					Selection (4*)	Comparability (3*)	Exposure (2*)
Piehl, 2017	39	39.6	27	35.2	**		***
Trentini, 2014	31	49.6	15	39	***		**
Novakova, 2017a	59	37	39 (dup)	33.6	****		***
Novakova, 2017b	43	39.7	39	33.6	****	*	***
Hakansson, 2017	22	unknown	22	32	****	**	****
Bergman, 2016	110	37.7	113	40.2	***		**
Lam, 2015	59	45.7	44	40.4	**		**
Stilund, 2015	59	41.2	39	40.7	***		**
Villar, 2015	127	33.6	37	34.6	**	*	***
Aeinehband, 2015	48	41.2	18	30.4	***		**
Burman, 2014	63	43.8	15	40.2	**		***
Axelsson, 2014	35	48	14	42	****	*	***
Fialová, 2013	18	38	24	33	**		**
Gunnarsson, 2011	92	37.3	28	43	***		**

Each study is scored using a star system based on three domains: (1) selection of study groups (cases and controls)—maximum 4*; (2) comparability of the groups—maximum 2*; (3) ascertainment of outcome—maximum 3*.

MS, multiple sclerosis; NOS, Newcastle-Ottawa Scale.

Seven studies were retrospective, four prospective and three were cross-sectional analyses. Seven studies referenced BioMS-EU guidelines, and two referenced guidelines relating to definition of control populations.^17 21 29^ All 14 studies used the commercially available Uman NfL ELISA to measure CSF NfL, with a lower limit of detection of 31 ng/L documented by the manufacturer, and intra-assay coefficient of variation reported by the authors between 3.5% and ‘<15%’. Seven papers explicitly reported that the analysis was blinded.^17 30–35^

### Statistical analysis

Standard mean deviation and 95% CIs were calculated for each group in each study. If only subgroup values were available from the datasets provided, means were combined and the SDs were pooled to get the cohort mean and SD.^36^ Where only the median and range were available, mean and SD were estimated using Luo *et al* 36 and Wan *et al*,^37^ respectively.^36 37^ If the paper provided data as SE of the mean, this too was converted to SD.

The individual means and SD were analysed in weighted fixed effect models to estimate standardised mean differences in NfL level between comparators (with 95% CI, and corresponding p value). Heterogeneity between studies was tested for, and documented as a Q test statistic and corresponding p value. Publication bias was assessed using funnel plots. Demographic differences between cohorts were tested for significance using two-way T-tests and Z-scores.

## Results

### CSF NfL in CDMS versus controls

**Figure 2:**
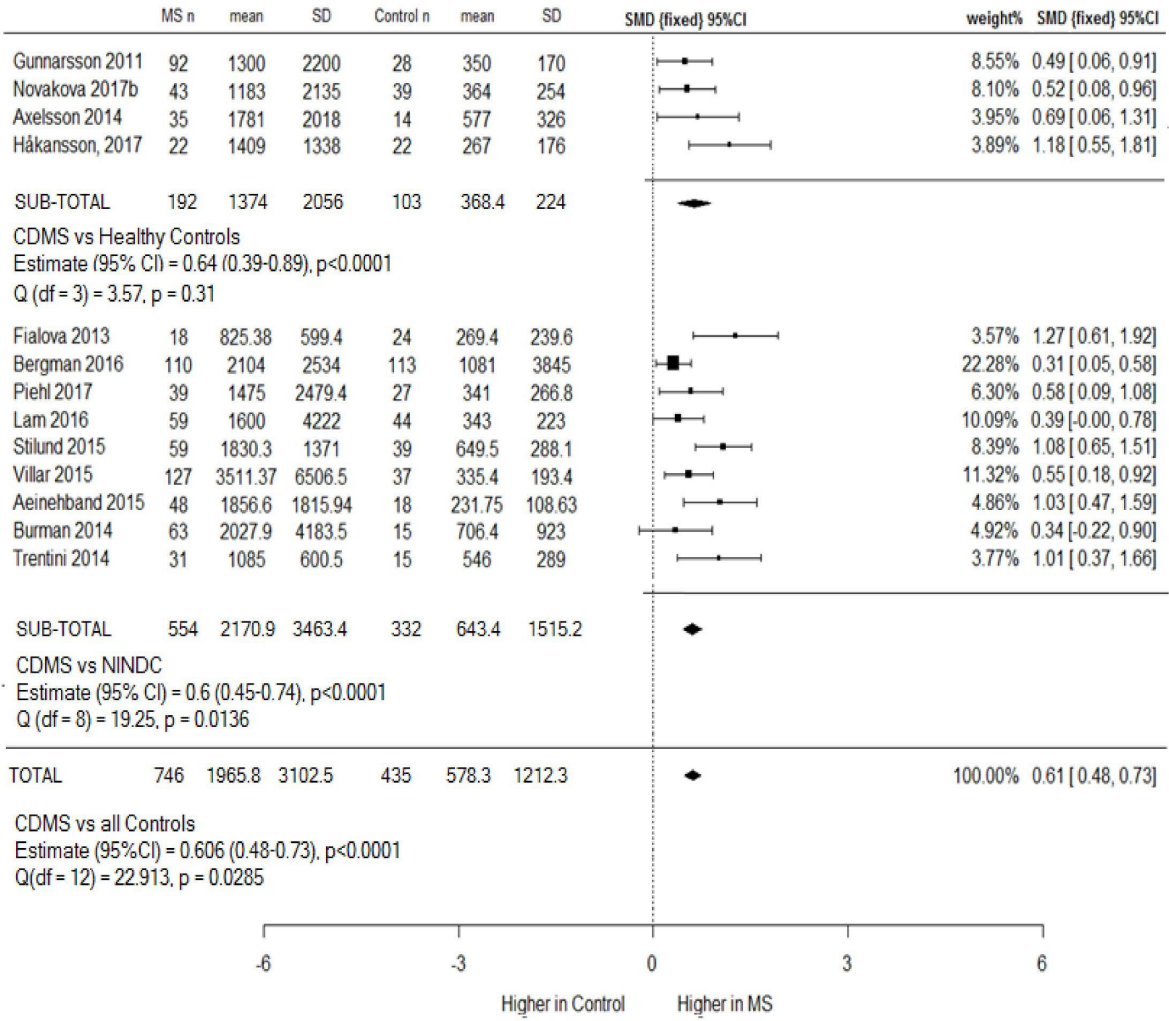
Clinically definite MS versus controls (HCs and NINDCs), subgroup and combined meta-analysis. Four studies compared CSF NfL levels in patients with MS with HCs, and nine studies used NINDCs. One group (Novakova *et al*) used the same control cohort in two papers (Novakova a and b). (Novakova a) was therefore excluded from the overall analysis to avoid duplication. This study is used in later subanalyses, and a sensitivity analysis including it did not alter results. CSF NfL levels are higher in MS than healthy and disease controls. CDMS, clinically definite MS; CSF, cerebrospinal fluid; HCs, healthy controls; MS, multiple sclerosis; NfL, neurofilament light chain; NINDCs, non-inflammatory neurological disease controls; SMD, standard mean deviation.

CSF NfL levels were higher in 746 patients with CDMS (1965.8 ng/L, SD=3102.5) than 435 controls (578.3 ng/L, SD=1212.3) (figure 2). MS and control groups were comparable in age (41.3 vs 37.3 years, respectively) and sex (63% and 62% women). Meta-analysis revealed a statistically significant moderate effect size, 0.61, p<0.00001. A funnel plot (online supplementary figure 2) showed spread around the observed outcome, making publication bias unlikely.

10.1136/jnnp-2018-319190.supp2 Supplementary data

The CSF NfL level in 332 NINDCs (643.4 ng/L, SD=1515.2) was double that seen in 103 HCs (368.4 ng/L, SD=224). Heterogeneity between study outcomes was significant in the CDMS versus NINDCs meta-analysis (p=0.0136), but not the CDMS versus HC meta-analysis. MS cohorts were demographically comparable (mean age 41.7 years; 56% or 65% women in the MS cohort compared with HC or NINDCs, respectively), with approximately a third of subjects in relapse. This suggests that differences between outcomes arise from differences between control populations.

### CSF NfL in RRMS versus controls

**Figure 3:**
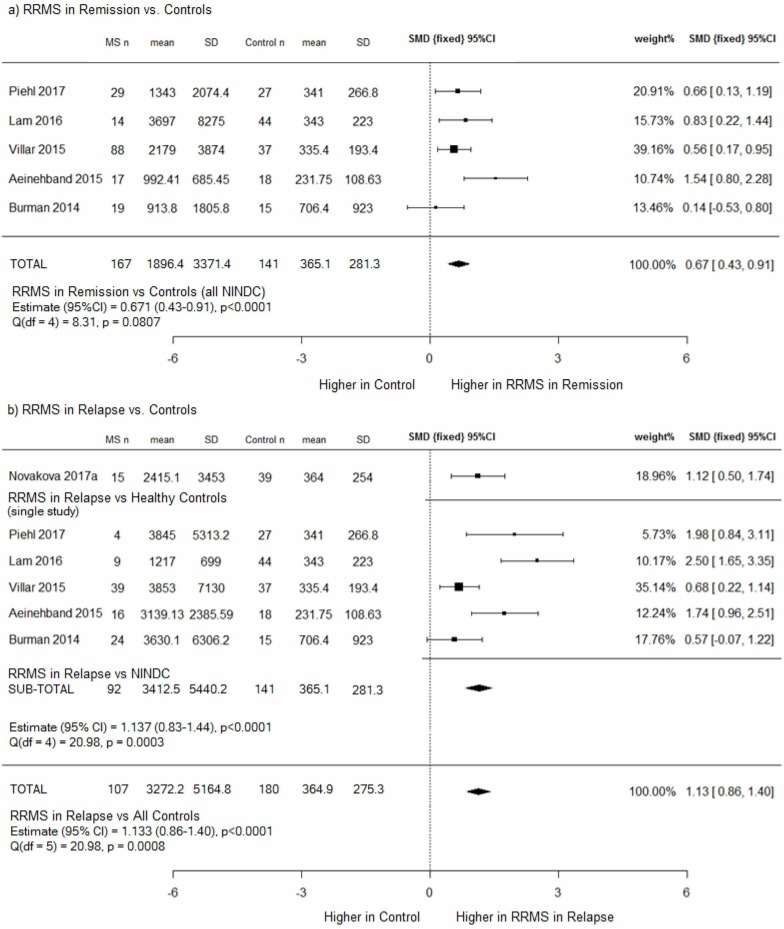
(A) RRMS in remission versus controls (NINDCs) and (b) RRMS in relapse versus controls (HCs and NINDCs), subgroup and combined meta-analysis. (a) CSF NfL levels were higher in patients with RRMS in remission than disease controls, with a moderate effect size of 0.67; (b) CSF NfL levels were higher in patients with RRMS in relapse than both healthy and disease controls, with a large effect size of 1.13; however, heterogeneity between studies was also significant. CSF, cerebrospinal fluid; HCs, healthy controls; NfL, neurofilament light chain; MS, multiple sclerosis; NINDCs, non-inflammatory neurological disease controls; RRMS, relapsing remitting MS; SMD, standard mean deviation.

CSF NfL is significantly higher in RRMS subjects (during both relapse and remission) than controls, p<0.00001 (figure 3). The effect size is larger during relapse (1.13) than remission (0.67).

In the ‘RRMS in remission’ versus controls (NINDCs) meta-analysis, no heterogeneity was evident, and the mean NfL level was five times higher in ‘RRMS in remission’ than NINDCs (1896.4 ng/L, SD=3371.4 versus 365.1 ng/L, SD=281.3). In the ‘RRMS in relapse’ versus controls meta-analysis, mean NfL was nine times higher in patients with RRMS (3272.2 ng/L, SD=5164.8 vs 364.9 ng/L, SD=275.3), but heterogeneity between study outcomes was significant, (p=0.0008).

As the same NINDCs were the comparator in both analyses, the ‘RRMS in relapse’ cohort is the source of the heterogeneity. Patients with RRMS ‘in relapse’ were 76% women, with a mean age of 35 years, whereas RRMS ‘in remission’ were 68% women, with a mean age of 38 years. Age and sex differences between the groups were not statistically significant (two-way T-test, p=0.104 and Z-score, p=0.37, respectively).

However, how the authors defined ‘relapse’, and thus ‘remission’, varied significantly (online supplementary file 3). Lam *et al* 38 defined their relapse cohort as those who had relapsed within 4 weeks of CSF sampling. Six studies included patients who had relapsed within 3 months (Piehl *et al*,^39^ Villar *et al*,^40^ Novakova *et al*,^41^ Gunnarsson *et al*,^31^ Axelsson *et al* 30 and Aeinehband *et al* 33), and Novakova *et al* 27 used <100 days. Burman *et al*^32^ defined a relapse as occurring within the preceding 3 months, or the presence of Gd +lesions on MRI, or in some patients a combination. The definition of disease state (relapse or remission) may therefore be the source of heterogeneity.

10.1136/jnnp-2018-319190.supp3 Supplementary data

### CSF NfL in relapse versus CSF NfL in remission

**Figure 4:**
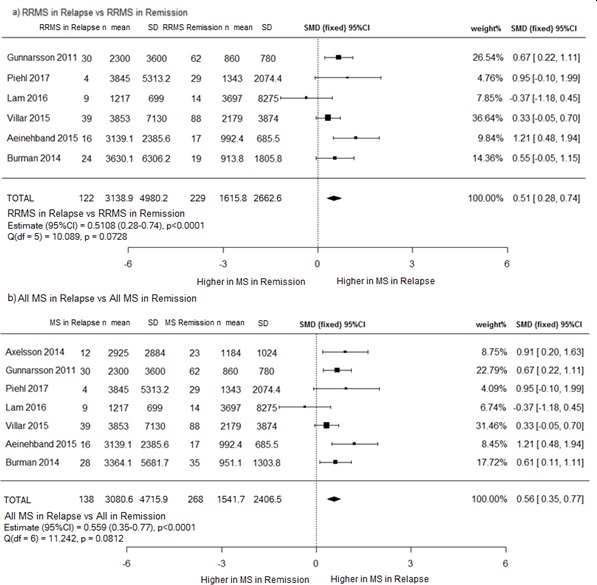
(A) RRMS in relapse versus RRMS in remission and (b) all patients with MS in relapse versus all MS. (a) CSF NfL levels are higher in RRMS in relapse than in remission; (b) when progressive and patients with RRMS are combined as ‘all patients’, CSF NfL levels remain higher in relapse patients than remission patients. CSF, cerebrospinal fluid; MS, multiple sclerosis; NfL, neurofilament light chain; RRMS, relapsing remitting MS; SMD, standard mean deviation.

The effect of relapse on CSF NfL level was similar irrespective of whether patients were defined as RRMS or progressive, with a moderate effect size seen in each (0.51 and 0.56, p<0.0001, respectively) (figure 4).

Mean CSF NfL in 122 patients with RRMS in relapse (3138.9 ng/L, SD 4980.2) was approximately twice that seen in 229 patients with RRMS in remission (1615.8 ng/L, SD 2662.6). When patients with progressive MS were included in the analysis, mean NfL values were similar (3080.6 ng/L, SD 4715.9 versus 1541.7 ng/L, SD 2406.5). Heterogeneity between studies was not significant for either analysis.

### CSF NfL in progressive MS

#### SPMS versus PPMS

Meta-analysis of 75 SPMS compared with 48 PPMS showed no difference in NfL levels (online supplementary figure 4). The populations appeared representative, and a funnel plot (online supplementary figure 5) showed no suggestion of publication bias. SPMS and PPMS were therefore combined for further analyses.

10.1136/jnnp-2018-319190.supp4 Supplementary data

10.1136/jnnp-2018-319190.supp5 Supplementary data

#### CSF NfL in progressive MS versus Controls

**Figure 5:**
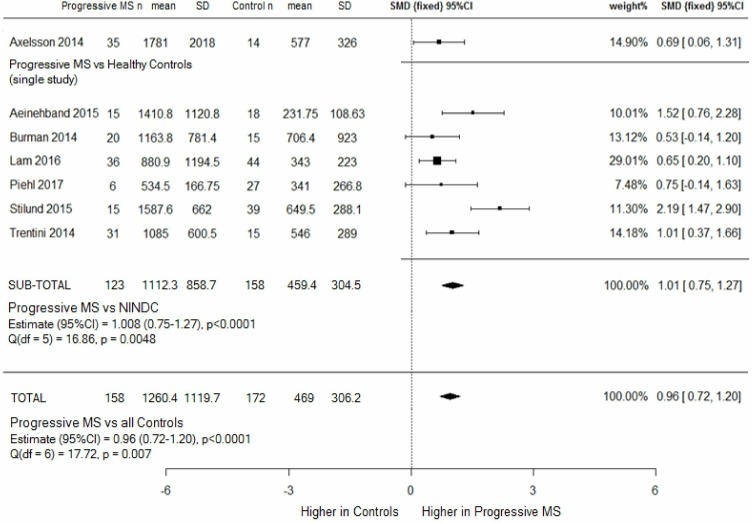
Progressive MS (combined SPMS and PPMS) versus controls (HC and NINDCs), subgroup and combined meta-analysis. CSF NfL levels were higher in patients with progressive disease than healthy and disease controls, but heterogeneity between studies was significant. HC=healthy controls; MS, multiple sclerosis; NINDCs, non-inflammatory neurological disease controls; PPMS, primary progressive MS; SMD, standard mean deviation; SPMS, secondary progressive MS.

Mean NfL levels were three times higher in 158 patients with progressive MS (1260.4 ng/L, SD=1119.7) than 158 NINDCs and 14 HCs (469 ng/L, SD=306.2), and meta-analysis showed a significant effect size of 0.96, p<0.00001 (figure 5). Patients with progressive MS were older (52.6 versus 38.4 years, p<0.001), and sex distribution was unequal (50.4% women vs 67.1% women, p=0.0047).

#### CSF NfL in RRMS versus progressive MS

**Figure 6:**
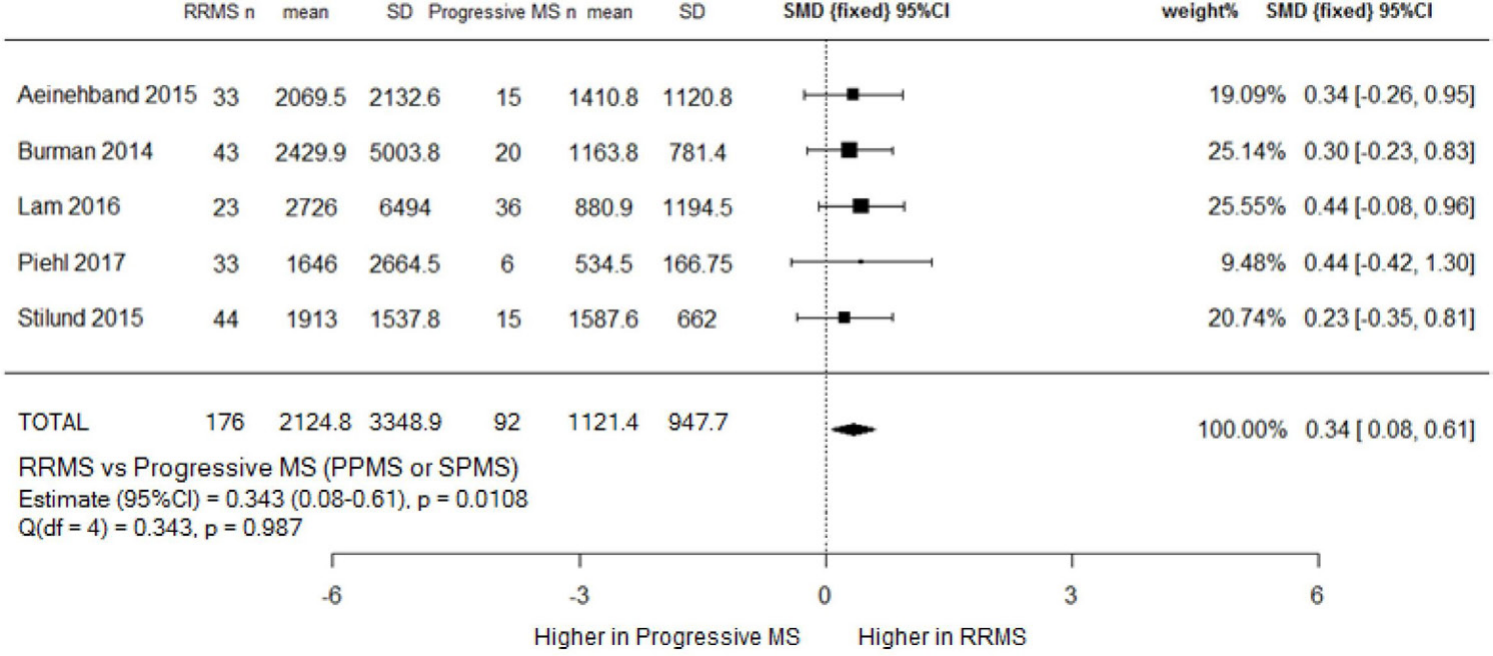
RRMS versus progressive MS (combined SPMS and PPMS) meta-analysis. CSF NfL levels were higher in RRMS than patients with progressive MS, with a small, but statistically significant, effect size of 0.34. PPMS, primary progressive MS; SMD, standard mean deviation; RRMS, relapsing remitting MS; SPMS, secondary progressive MS.

Meta-analysis of five studies showed a higher CSF NfL in 176 patients with RRMS compared with 92 patients with progressive MS (2124.8 ng/L vs 1121.4 ng/L) (figure 6). The effect size was small (0.34), but statistically significant, p=0.0108.

Demographic data were available for 87% of all subjects in this analysis. Patients with RRMS were younger (40.1 years vs 53.4 years), with a greater proportion of women (74% vs 53%). 18% of RRMS, but only 8% of patients with progressive MS, were on DMT. Relapse data were available for 75% of the RRMS cohort (of which 40.2% were in relapse) and 83.7% of the progressive cohort (of which 5.2% were in relapse). There was a marked difference in disease duration between the cohorts—69.9 months for patients with RRMS compared with 167.5 months for patients with progressive MS. Limited subgroup data prevented us from being able to analyse the relationship between disease duration and NfL levels. Within the progressive cohort, we did however note that patients with SPMS had a longer mean disease duration than patients with PPMS (204.3 vs 59.5 months), and that mean CSF NfL levels between the two did not differ.

### CSF NfL level in treated and untreated MS

Meta-analysis of 163 treated and 70 untreated patients with MS showed no effect of treatment on CSF NfL level (online supplementary figure 6). The majority (78%) of patients were treated with low efficacy DMTs—interferon-beta, glatiramer acetate or teriflunomide, and 2% were treated with intravenous immunoglobulin. A minority were taking high efficacy DMT (10% natalizumab and 6% mitoxantrone). In 4% therapy was recorded as ‘other’.^32^ There was no evidence of publication bias (online supplementary figure 7). As expected, heterogeneity between study outcomes was significant.

10.1136/jnnp-2018-319190.supp6 Supplementary data

10.1136/jnnp-2018-319190.supp7 Supplementary data

## Discussion

NfL has been proposed both as a standalone biomarker of neurodegeneration and as a component of a composite ‘treatment target’ measure (no evidence of disease activity−5).^42^ The role of NfL levels in clinical practice (as a measure of neurodegeneration or inflammatory activity, as a tool to monitor DMT efficacy or as a prognostic biomarker) is yet to be determined, but the ability to measure blood levels increases their clinical utility considerably. We chose to perform a systematic review of NfL in CSF because, due to the proximity to the pathology, CSF NfL levels may more accurately reflect axonal injury than blood levels, and are less likely to be influenced by factors such as blood–brain-barrier integrity or systemic comorbidities.

### CSF NfL in CDMS

CSF NfL levels were approximately three times higher in patients with CDMS than controls; however, heterogeneity between study outcomes limits our ability to suggest ‘standard’ or ‘expected’ CSF NfL levels.

Heterogeneity appeared to arise from NINDC populations, which had a mean NfL level double that of HC, but with a SD six times greater. This is not unexpected given that NfL is a non-specific biomarker of axonal damage. However, such a diverse control population is problematic when comparing results or combining datasets.

The two groups in this meta-analysis that referenced BioMS-EU guidelines on defining control populations used ‘symptomatic controls’ (Stilund *et al* 29) and HCs (Trentini *et al* 17). Seven papers provided details of 177 (53%) NINDCs (online supplementary figure 1). Two studies included patients with idiopathic intracranial hypertension (IIH) (Villar *et al* 40 and Burman *et al*,^32^ who noted NINDCs with markedly elevated NfL levels had a diagnosis of IIH). IIH has been associated with high CSF NfL levels and is frequently included in NINDC populations.^43^

Six of the nine studies using NINDCs explicitly reported a normal CSF cell count and IgG index/OCB status. One paper (Lam *et al* 38) included 2/45 NINDCs in whom OCBs were detected in the CSF. Two studies reported NINDCs had normal MRI scans,^29 33^ three reported MRI showed ‘no inflammation’ or ‘no features of MS’,^32 38 44^ and four did not comment.

Data were not available to characterise 155 NINDCs across three studies. All three studies reported normal CSF findings, and an absence of inflammation on MRI scans. Clinical examination was not recorded for 116 NINDCs, and was recorded as ‘normal clinical data’ in 39. NfL levels are increased in diseases such as Parkinson’s disease and amyotrophic lateral Sclerosis, where CSF and MRI findings can be normal. CSF and MRI alone therefore do not exclude conditions which may result in elevated NfL levels. Routine use of guidelines on defining control populations may enable datasets to be more easily combined.

### CSF *NfL in relapse*

Compared with controls, patients with RRMS had neurofilament levels five times higher during remission, and nine times higher during relapse, replicating previous findings.^25^ Significant heterogeneity within the RRMS relapsing population warrants caution in transcribing our findings to an individual patient level. The source of heterogeneity here lies in the definition of ‘relapse’. Most studies defined relapse populations as those with a clinical relapse within the 3 months prior to CSF sampling. However, CSF NfL increases acutely in the context of relapse, peaking around 3 weeks, and remaining elevated for more than 15 weeks.^25 45^ The half-life of CSF and serum NfL is unknown, and none of the studies included in this meta-analysis were designed to assess the temporal relationship between NfL levels and relapses. Lam *et al* 38 defined their relapse cohort as relapsing within 4 weeks of CSF sampling, so sampling ‘too early’ may explain why the lowest mean NfL (and SD) was seen in this study.

Only cases that were explicitly stated to be in remission were included in remission groups for this meta-analysis, but by groups excluding only those with clinical relapse (rather than radiological evidence of activity) NfL levels may be falsely elevated in the ‘remission’ cohorts. Burman *et al* 32 included patients with radiological findings of relapse (with or without clinical relapse) in their relapse cohort, and recorded the highest mean CSF NfL (and SD).

Longitudinal NfL analysis within individual patients is required, but has, until recently, been impractical due to the invasive nature of sampling. Technological advances now allow measurement of NfL at femtolitre concentrations using a single molecule array ELISA. This enables NfL levels to be measured in blood, and early studies suggest a good correlation between CSF and blood NfL levels of r=0.77 - r=0.97.^41 46^ Studies reviewing the temporal relationship between NfL levels and relapse are currently lacking. Future studies examining this might inform the rate of NfL normalisation after relapse, and if NfL levels peak/fall more rapidly in different disease subgroups, or in the context of varying degrees of clinical recovery.

To assess the effect of relapse on NfL levels in progressive MS, we went on to include patients with progressive MS in this analysis. A statistically significant moderate effect size favouring those in relapse remained, suggesting a potential role of CSF NfL in quantification of relapsing activity across all MS subtypes. As DMTs become available for progressive MS, the ability to identify markers of active inflammatory disease may play an important role in decisions regarding treatment options and in monitoring.

### CSF NfL in progressive MS

CSF NfL levels in patients with progressive MS were twice that of controls (although confounders were evident), but significantly lower than in patients with RRMS (relapse and remission). Heterogeneity reflects the expected differences in demographics and disease activity between the cohorts, and although the RRMS studies with the highest proportion of patients in relapse did not have the highest mean NfL, the effect of relapses is reflected by the lower SD in the progressive cohort compared with the RRMS cohort.

To assess whether CSF NfL was significantly different between RRMS in remission and progressive disease, we compared data from 229 patients with RRMS in remission with 158 patients with progressive MS. Patients with RRMS in remission had a higher mean NfL level (1615.8 ng/L, SD=2662.6 vs 1260.4 ng/L, SD=1119.7), but was not statistically significant (p=0.072). The trend in these data is consistent with the hypothesis that intermittent, inflammatory, disease activity might have a more pronounced effect on CSF NfL level than cumulative neuroaxonal loss. However, a purely clinical definition of remission may have missed subclinical relapses, resulting in a falsely high CSF NfL in the remission cohort.

### CSF NfL and DMT

High efficacy DMTs (such as Natalizumab and Fingolimod) are associated with reductions in NfL level post-treatment, irrespective of clinical course, and independent of relapse rate, to the point that levels are not significantly higher than in HCs.^30 31 41 47 49^ Our meta-analysis however showed no effect of DMT on CSF NfL levels. This may be because the our numbers in this analysis were small, and the majority of our cohorts were treated with less efficacious agents, which are known to reduce relapses to a lesser degree.

Limitations of our meta-analysis include the fact that a single author reviewed the literature, and that studies were excluded due to insufficient data. We contacted the authors, but had to exclude six papers, and in two papers had to estimate the mean and SD using the median and range. A further limitation was that we used raw data (uncorrected for age). The reason for this was that only some studies corrected data for age (when analysing results), and in others the demographic data provided were not sufficiently detailed to allow correction for age within subgroups. Studies that analysed correlation between NfL level and age showed mixed results. Four studies found no correlation (17 34 40 48). Two found a positive correlation in control populations.^32 33^ Four papers reported a positive correlation in patients with MS, but only one was statistically significant (r=0.216, p<0.0008).^40^ Others reported ‘age dependency’ and adjusted their analyses.^27 44^ Overall, literature suggests a positive correlation between age and CSF NfL level. However, the small effect of age on NfL levels is probably masked by higher disease-associated influences on NfL levels in the young MS population.

This meta-analysis provides evidence that CSF neurofilament levels are significantly higher in pwMS than controls, and in pwMS in relapse compared with remission, regardless of MS subtype. Raised CSF NfL levels in all pwMS suggest that axonal damage occurs throughout the disease course, and not simply in the context of relapses, or as a late phenomenon. CSF NfL levels do not clearly distinguish MS subtypes, and NfL is not a useful tool for ‘staging’ MS. In all patients, relapse appears to be a stronger driver of NfL levels than progressive disease. This suggests that CSF NfL correlates more closely with acute inflammation than chronic neurodegeneration, and that NfL may have greater clinical utility as a biomarker of disease activity than disease progression.

There is now unequivocal data supporting a high correlation between CSF and blood NfL levels. In this new era of ultra-high sensitivity biomarkers, we are moving from CSF to serum-based assays, which offer advantages such as longitudinal analysis. This is therefore an appropriate time to systematically evaluate the literature on CSF NfL in order to direct future studies.

In summary, we have shown that CSF NfL levels are higher in all subtypes of MS compared with healthy and disease controls. Furthermore we have shown that CSF NfL levels correlate most closely with inflammatory disease activity.

## References

[1] Thompson AJ, Banwell BL, Barkhof F, et al Diagnosis of multiple sclerosis: 2017 revisions of the McDonald criteria. Lancet Neurol 2018;17:162–73. 10.1016/S1474-4422(17)30470-2 29275977

[2] Miller DH Magnetic resonance in monitoring the treatment of multiple sclerosis. Ann Neurol 1994;36 Suppl:S91–S94. 10.1002/ana.410360720 8017895

[3] Arrambide G, Rovira A, Sastre-Garriga J, et al Spinal cord lesions: a modest contributor to diagnosis in clinically isolated syndromes but a relevant prognostic factor. Mult Scler J [Internet] 2017;1352458517697830. 10.1177/1352458517697830 28301287

[4] Zivadinov R, Stosic M, Cox JL, et al The place of conventional MRI and newly emerging MRI techniques in monitoring different aspects of treatment outcome. J Neurol 2008;255:61–74. 10.1007/s00415-008-1009-1 18317678

[5] Thompson AJ, Hobart JC Multiple sclerosis: assessment of disability and disability scales. J Neurol 1998;245:189–96. 10.1007/s004150050204 9591219

[6] Filippi M, Paty DW, Kappos L, et al Correlations between changes in disability and T2-weighted brain MRI activity in multiple sclerosis: a follow-up study. Neurology 1995;45:255–60. 10.1212/WNL.45.2.255 7854522

[7] Li DKB, Held U, Petkau J, et al MRI T2 lesion burden in multiple sclerosis: a plateauing relationship with clinical disability. Neurology 2006;66:1384–9. 10.1212/01.wnl.0000210506.00078.5c 16682671

[8] Anderson VM, Fox NC, Miller DH Magnetic resonance imaging measures of brain atrophy in multiple sclerosis. J Magn Reson Imaging 2006;23:605–18. 10.1002/jmri.20550 16596564

[9] De Stefano N, Narayanan S, Francis GS, et al Evidence of axonal damage in the early stages of multiple sclerosis and its relevance to disability. Arch Neurol 2001;58:65–70. 10.1001/archneur.58.1.65 11176938

[10] Fisher E, Rudick RA, Cutter G, et al Relationship between brain atrophy and disability: an 8-year follow-up study of multiple sclerosis patients. Mult Scler 2000;6:373–7. [Internet] 10.1177/135245850000600602 11212131

[11] De Stefano N, Battaglini M, Smith SM Measuring brain atrophy in multiple sclerosis. J Neuroimaging 2007;17(SUPPL. 1):10S–15. 10.1111/j.1552-6569.2007.00130.x 17425728

[12] Lobsiger CS, Cleveland DW Neurofilaments: Organization and Function in Neurons : Encyclopedia of neuroscience. In, 2010: 433–6 p..

[13] Ibl G NF-light (neurofilament light). IBL NF-L ELISA 2015;49:1–7.

[14] Petzold A, Altintas A, Andreoni L, et al Neurofilament ELISA validation. J Immunol Methods 2010;352:23–31. [Internet] 10.1016/j.jim.2009.09.014 19857497

[15] C-H L, Macdonald-Wallis C, Gray E, et al Neurofilament light chain: a prognostic biomarker in amyotrophic lateral sclerosis. Neurology 2015;84:2247–57. [Internet]. 25934855 10.1212/WNL.0000000000001642 PMC4456658

[16] Soelberg Sorensen P, Sellebjerg F Neurofilament in CSF—A biomarker of disease activity and long-term prognosis in multiple sclerosis. Mult Scler 2016;22:1112–3. 10.1177/1352458516658560 27364323

[17] Trentini A, Comabella M, Tintoré M, et al N-acetylaspartate and neurofilaments as biomarkers of axonal damage in patients with progressive forms of multiple sclerosis. J Neurol 2014;261:2338–43. [Internet] 10.1007/s00415-014-7507-4 25228004

[18] Kuhle J, Barro C, Disanto G, et al Serum neurofilament light chain in early relapsing remitting MS is increased and correlates with CSF levels and with MRI measures of disease severity. Mult Scler 2016;22:1550–9. 10.1177/1352458515623365 26754800

[19] Lublin FD, Reingold SC, CohenJa , et al Defining the clinical course of multiple sclerosis : The 2013 revisions Defining the clinical course of multiple sclerosis The 2013 revisions. Neurology 2014;83:278–86. 24871874 10.1212/WNL.0000000000000560 PMC4117366

[20] Teunissen CE, Petzold A, Bennett JL, et al A consensus protocol for the standardization of cerebrospinal fluid collection and biobanking. Neurology 2009;73:1914–22. 10.1212/WNL.0b013e3181c47cc2 19949037 PMC2839806

[21] Teunissen C, Menge T, Altintas A, et al Consensus definitions and application guidelines for control groups in cerebrospinal fluid biomarker studies in multiple sclerosis. Mult Scler 2013;19:1802–9. 10.1177/1352458513488232 23695446

[22] McDonald WI, Compston A, Edan G, et al Recommended diagnostic criteria for multiple sclerosis: guidelines from the International panel on the diagnosis of multiple sclerosis. Ann Neurol. 2001;50:121–7. 10.1002/ana.1032 11456302

[23] Polman CH, Reingold SC, Banwell B, et al Diagnostic criteria for multiple sclerosis: 2010 revisions to the McDonald criteria. Ann Neurol. 2011;69:292–302. 10.1002/ana.22366 21387374 PMC3084507

[24] Moher D PRISMA 2009 checklist PRISMA 2009 checklist. Vol. 6. PLoS medicine 2009:e1000097.19621072

[25] Malmeström C, Haghighi S, Rosengren L, et al Neurofilament light protein and glial fibrillary acidic protein as biological markers in MS. Neurology 2003;61:1720–5. [Internet] 10.1212/01.WNL.0000098880.19793.B6 14694036

[26] Norgren N, Sundström P, Svenningsson A, et al Neurofilament and glial fibrillary acidic protein in multiple sclerosis. Neurology 2004;63:1586–90. [Internet] 10.1212/01.WNL.0000142988.49341.D1 15534240

[27] Novakova L, Axelsson M, Khademi M, et al Cerebrospinal fluid biomarkers as a measure of disease activity and treatment efficacy in relapsing-remitting multiple sclerosis. J. Neurochem. 2017;141:296–304. 10.1111/jnc.13881 27787906

[28] Stang A Critical evaluation of the Newcastle-Ottawa scale for the assessment of the quality of nonrandomized studies in meta-analyses. Eur J Epidemiol 2010;25:603–5. 10.1007/s10654-010-9491-z 20652370

[29] Stilund M, Gjelstrup MC, Petersen T, et al Biomarkers of inflammation and axonal Degeneration/Damage in patients with newly diagnosed multiple sclerosis: contributions of the soluble CD163 CSF/Serum ratio to a biomarker panel. Plos One 2015;10:e0119681 10.1371/journal.pone.0119681 25860354 PMC4393241

[30] Axelsson M, Malmeström C, Gunnarsson M, et al Immunosuppressive therapy reduces axonal damage in progressive multiple sclerosis. Mult Scler 2014;20:43–50. [Internet] 10.1177/1352458513490544 23702432

[31] Gunnarsson M, Malmeström C, Axelsson M, et al Axonal damage in relapsing multiple sclerosis is markedly reduced by natalizumab. Ann Neurol. 2011;69:83–9. [Internet] 10.1002/ana.22247 21280078

[32] Burman J, Zetterberg H, Fransson M, et al Assessing tissue damage in multiple sclerosis: a biomarker approach. Acta Neurol Scand 2014;130:81–9. [Internet] 10.1111/ane.12239 24571714

[33] Aeinehband S, Lindblom RPF, Al Nimer F, et al Complement component C3 and butyrylcholinesterase activity are associated with neurodegeneration and clinical disability in multiple sclerosis. Plos One 2015;10:e0122048 [Internet] 10.1371/journal.pone.0122048 25835709 PMC4383591

[34] Håkansson I, Tisell A, Cassel P, et al Neurofilament light chain in cerebrospinal fluid and prediction of disease activity in clinically isolated syndrome and relapsing-remitting multiple sclerosis. Eur J Neurol 2017;24:703–12. 10.1111/ene.13274 28261960

[35] Cohen J Statistical power analysis for the behavioral sciences. 2nd Edition, 1988.

[36] Luo D Xiang Wan JL and TT. optimally estimating the sample Mean from the sample size, median, mid-range and/or mid-quartile range. Statistical Methods in Medical Research 2017. 10.1177/0962280216669183 27683581

[37] Wan X, Wang W, Liu J, et al Estimating the sample mean and standard deviation from the sample size, median, range and/or interquartile range. BMC Med Res Methodol 2014;14 10.1186/1471-2288-14-135 PMC4383202 25524443

[38] Lam MA, Maghzal GJ, Khademi M, et al Absence of systemic oxidative stress and increased CSF prostaglandin F _2α_ in progressive MS. Neurol Neuroimmunol Neuroinflamm 2016;3:e256–9. 10.1212/NXI.0000000000000256 27386506 PMC4929888

[39] Piehl F, Kockum I, Khademi M, et al Plasma neurofilament light chain levels in patients with MS switching from injectable therapies to fingolimod. Mult Scler J [Internet] 2017;1352458517715132. 10.1177/1352458517715132 28627962

[40] Villar LM, Picón C, Costa-Frossard L, et al Cerebrospinal fluid immunological biomarkers associated with axonal damage in multiple sclerosis. Eur J Neurol 2015;22:1169–75. 10.1111/ene.12579 25324032

[41] Novakova L NFL in serum ECTRIMS Abstract- P1130 2017.

[42] Giovannoni G, Davorka T, Jeremy B, et al No evident disease activity. Mult Scler J 2017;23:1179–87. 10.1177/1352458517703193 PMC5536258 28381105

[43] Ågren-Wilsson A, Lekman A, Sjöberg W, et al CSF biomarkers in the evaluation of idiopathic normal pressure hydrocephalus. Acta Neurol Scand 2007;116:333–9. 10.1111/j.1600-0404.2007.00890.x 17922727

[44] Bergman P, Piket E, Khademi M, et al Circulating miR-150 in CSF is a novel candidate biomarker for multiple sclerosis. Neurology - Neuroimmunology Neuroinflammation 2016;3:e219 10.1212/NXI.0000000000000219 PMC4841644 27144214

[45] Giovannoni G Cerebrospinal fluid neurofilament: the biomarker that will resuscitate the ‘Spinal Tap’. Mult Scler 2010;16:285–6. 10.1177/1352458510361358 20203146

[46] Disanto G, Barro C, Benkert P, et al Serum neurofilament light: a biomarker of neuronal damage in multiple sclerosis. Ann Neurol 2017;81:857–70. 10.1002/ana.24954 28512753 PMC5519945

[47] Kuhle J, Disanto G, Lorscheider J, et al Fingolimod and CSF neurofilament light chain levels in relapsing-remitting multiple sclerosis. Neurology 2015;84:1639–43. [Internet] 10.1212/WNL.0000000000001491 25809304 PMC4409586

[48] Fialová L, Bartos A, Svarcová J, et al Serum and cerebrospinal fluid light neurofilaments and antibodies against them in clinically isolated syndrome and multiple sclerosis. J Neuroimmunol 2013;262:113–20. 10.1016/j.jneuroim.2013.06.010 23870535

[49] Novakova L, Axelsson M, Khademi M, et al Cerebrospinal fluid biomarkers of inflammation and degeneration as measures of fingolimod efficacy in multiple sclerosis. Mult Scler J [Internet] 2016:1–10. 10.1177/1352458516639384 27003946

