# Supplementary material for: Cerebrospinal fluid neurofilament light chain in multiple sclerosis and its subtypes: a meta-analysis of case–control studies
Source: J Neurol Neurosurg Psychiatry. 2019 May 23;90(9):1059–67. doi: 10.1136/jnnp-2018-319190 (PMC6820150; doi:10.1136/jnnp-2018-319190)
Supplement: Supplementary data [file jnnp-2018-319190supp008.pdf]

1. Martin SJ

CSF Neurofilament light chain in multiple sclerosis and its subtypes: a meta-analysis of case control studies

Supplementary data Tables and Figures

Table 1 - Search Strategy

Table 2 - Inclusion / Exclusion Criteria

Table 3 - Reasons for exclusion

2. Martin SJ

Search Term	Search Number	Number of Hits (08/09/2017)
‘MS’ OR ‘Multiple sclerosis’	1	Pubmed – 303,063
		Web of Science – 307,800
		Cochrane Library – 14,503
		OpenGrey database– 3,044
‘NFL’ OR ‘Neurofilament light’	2	Pubmed – 1,456
		Web of Science – 1,673
		Cochrane Library - 84
		OpenGrey database- 1
‘CSF’ OR ‘Cerebrospinal fluid’	3	Pubmed – 132,444
		Web of Science – 60,522
		Cochrane Library – 7,220
		OpenGrey database- 337
1 AND 2 AND 3	4	**Pubmed - 83**
		**Web of Science - 124**
		**Cochrane Library - 12**
		**OpenGrey database - 0**
	Total Number of Hits	**= 219**

Supplementary Data Table 1: Search Strategy

3. Martin SJ

**Inclusion Criteria**	**Exclusion Criteria**
Diagnosis of MS meeting established diagnostic criteria	Clinically or radiologically isolated syndrome
Case control studies (retrospective or prospective) producing original work.	Animal studies, reviews or responses, or manuscripts unrelated to research topic.
Neurofilament light measured in CSF in quantitative manner.	NfL levels detectable in less than 85% of either MS or control cohort.
CSF biobanking referencing established guidelines, or if not, ELISA protocol described.	Coefficient of Variation >25%
Validated assay or, if not, description of ELISA technique and lower limit of detection	NfL data given as relative units or not absolute values.

Supplementary Data Table 2: Inclusion / Exclusion Criteria

4. Martin SJ

Supplementary Data Table 3: Reasons for exclusion

	**Reason for exclusion**	**Number**
**Excluded on Abstract**	Not Multiple Sclerosis	47
	Summary or Review article	23
	Basic science study/Animal (not clinical)	14
	NfL AB or other measured	9
	Assay analytical analysis or validation	4
	Only abstract available	3
	**Total**	**100**
**Excluded on Full text**	No control group	17
	Assay sens <85% for MS/ control group	7
	NfL cohort data published elsewhere	3
	Semi-quantitative data	3
	NfL measured only in serum	1
	**Total**	**31**
**Excluded as required dataset unavailable**		**6**
**Raw data or dataset available for inclusion in meta-analysis**		**14**

5. Martin SJ
