# Supplementary figures and images for: Cerebrospinal fluid neurofilament light chain in multiple sclerosis and its subtypes: a meta-analysis of case–control studies
Source: J Neurol Neurosurg Psychiatry. 2019 May 23;90(9):1059–67. doi: 10.1136/jnnp-2018-319190 (PMC6820150; doi:10.1136/jnnp-2018-319190)

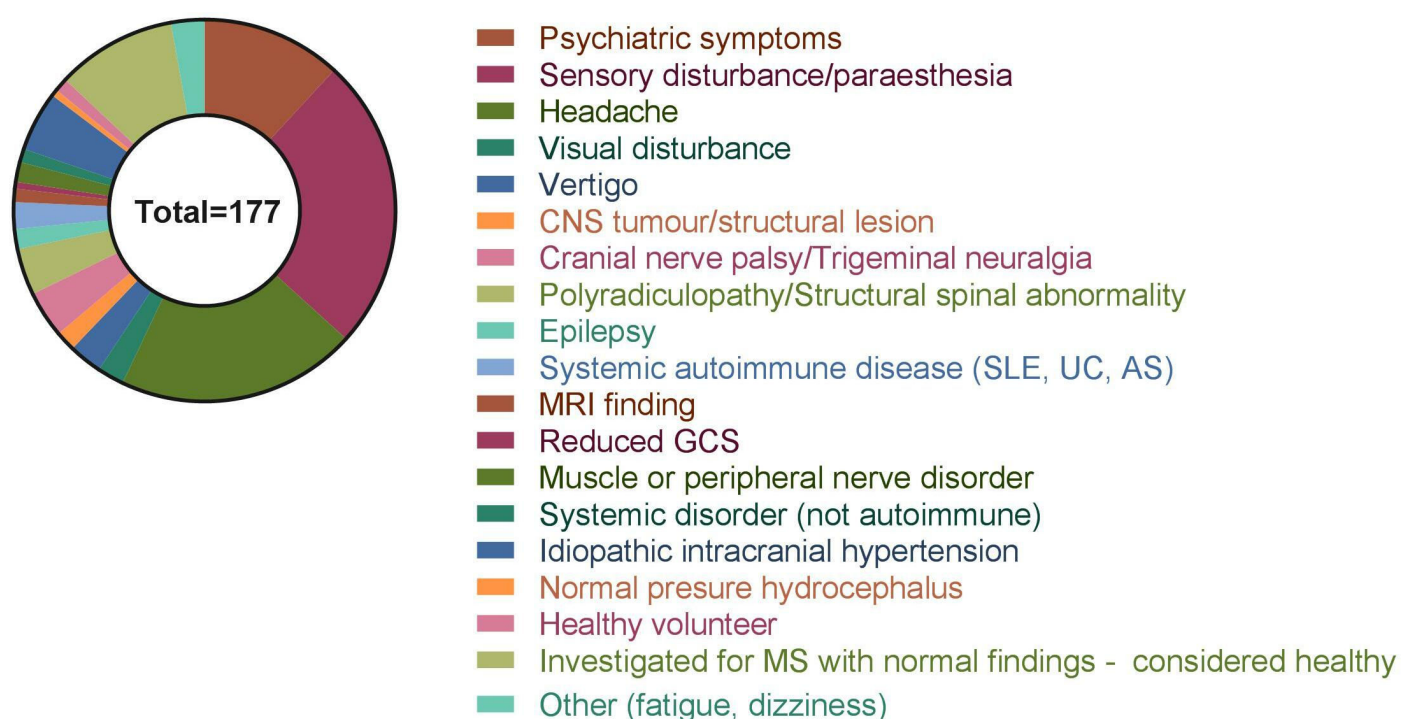

Supplement: Supplementary data [file jnnp-2018-319190supp001.pdf]

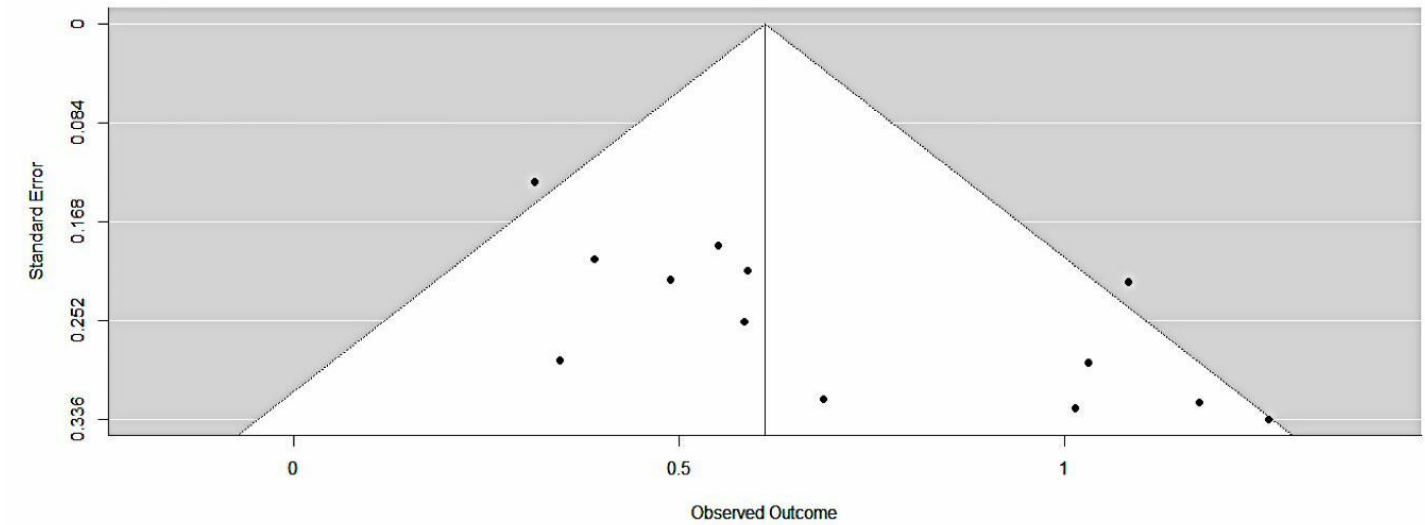

Supplement: Supplementary data [file jnnp-2018-319190supp002.pdf]

## Maximum number of days between clinical relapse and CSF sampling

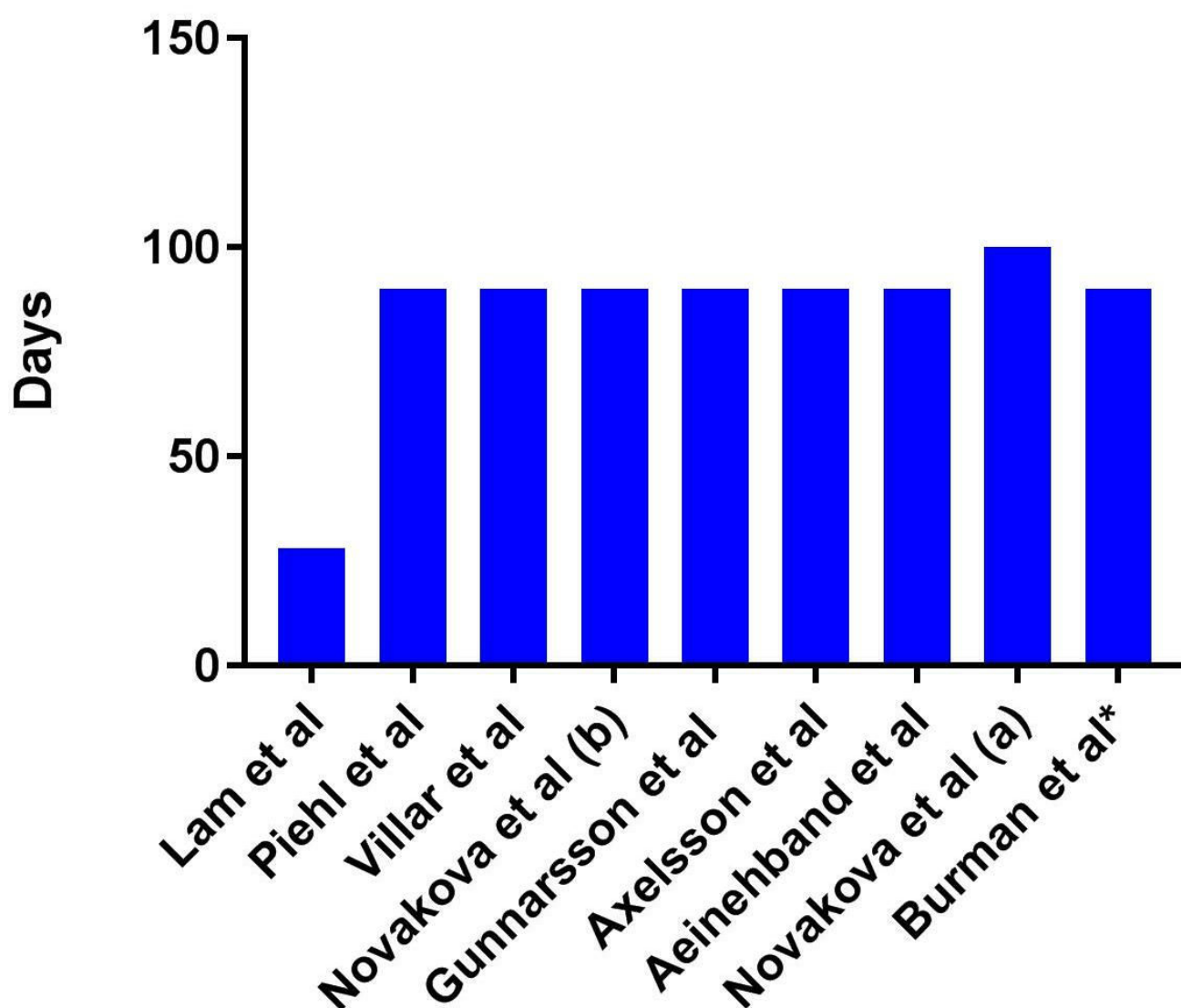

Supplement: Supplementary data [file jnnp-2018-319190supp003.pdf]

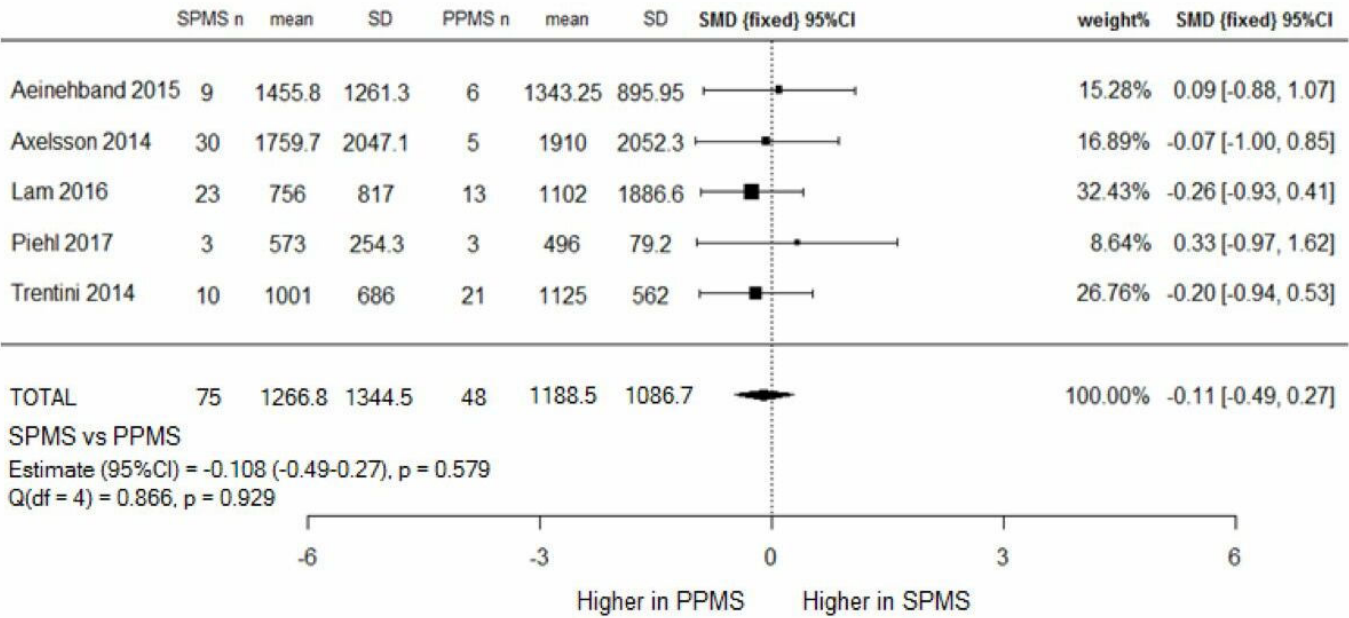

Supplement: Supplementary data [file jnnp-2018-319190supp004.pdf]

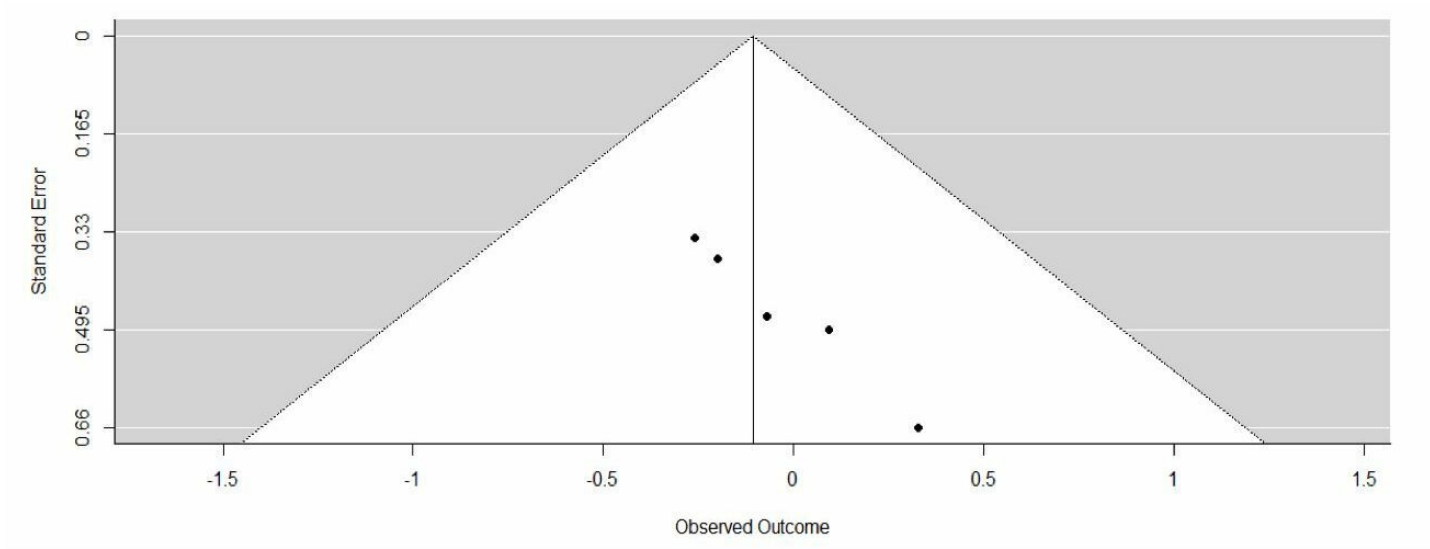

Supplement: Supplementary data [file jnnp-2018-319190supp005.pdf]

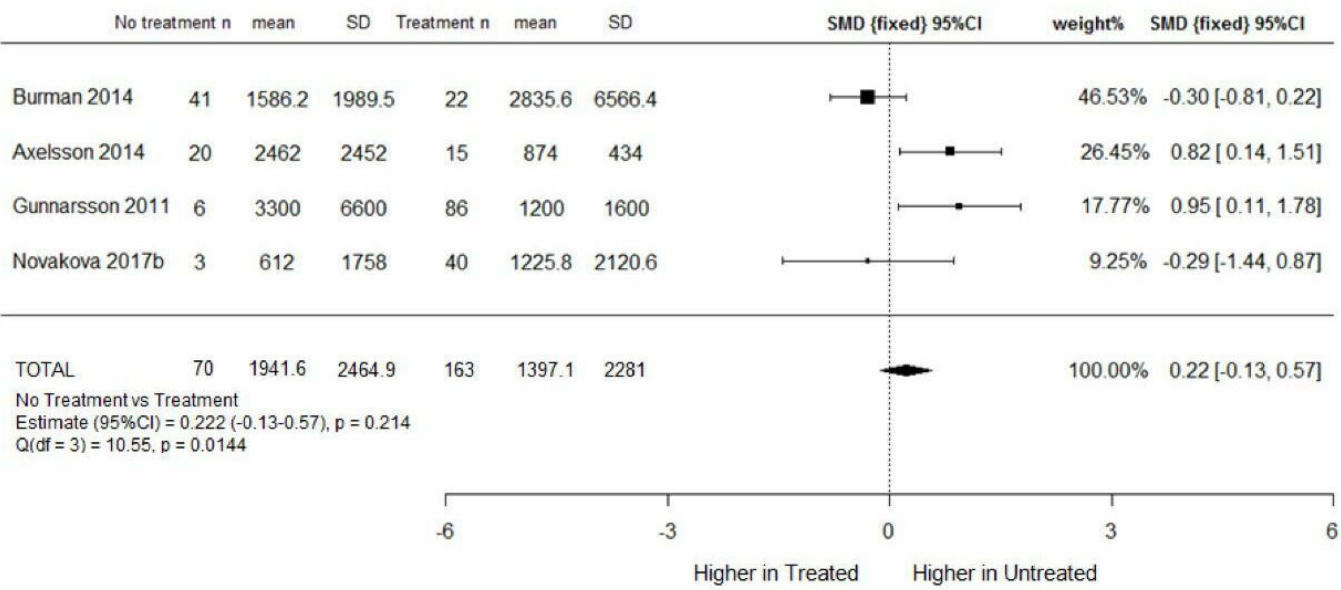

Supplement: Supplementary data [file jnnp-2018-319190supp006.pdf]

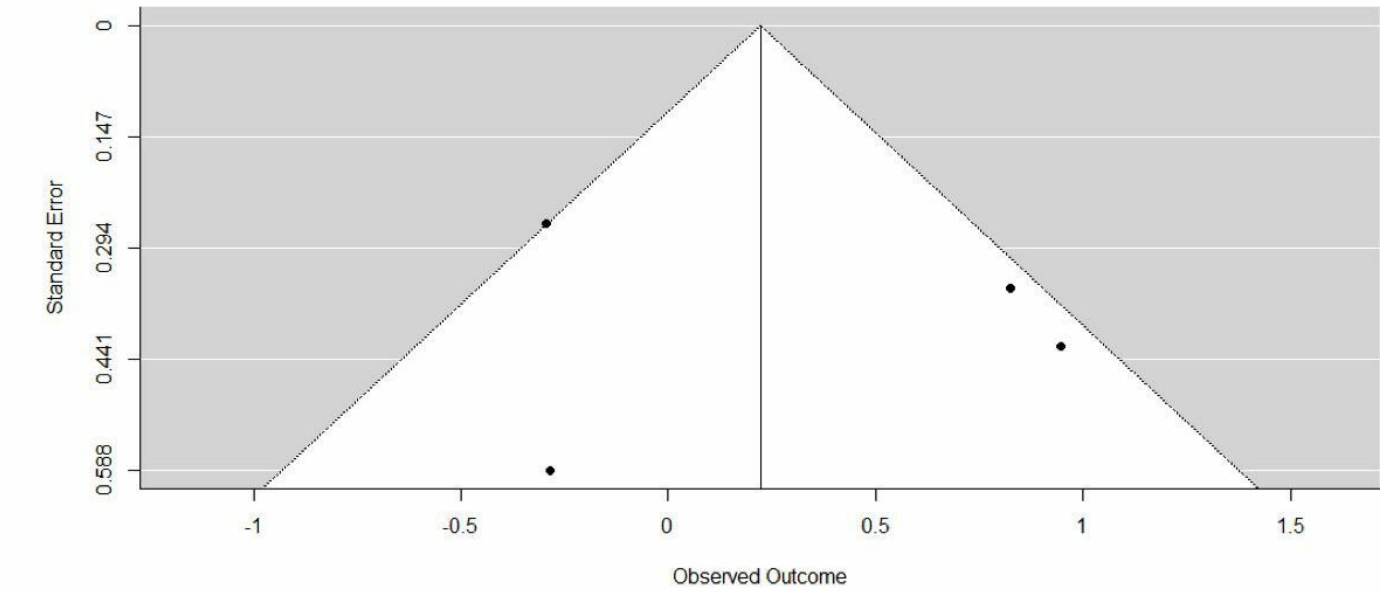

Supplement: Supplementary data [file jnnp-2018-319190supp007.pdf]
